# The Efficacy and Safety of Revefenacin for the Treatment of Chronic Obstructive Pulmonary Disease: A Systematic Review

**DOI:** 10.3389/fphar.2021.667027

**Published:** 2021-10-20

**Authors:** Jiaxing Zhang, Yihong Xie, Joey Sum-wing Kwong, Long Ge, Rui He, Wenyi Zheng, Jing Han, Rui Zhang, Huaye Zhao, Yuru He, Xiaosi Li

**Affiliations:** ^1^ Department of Pharmacy, Guizhou Provincial People’s Hospital, Guiyang, China; ^2^ Department of Pharmacy, Hospital of Chengdu Office of People’s Government of Tibetan Autonomous Region, Chengdu, China; ^3^ Global Health Nursing, Graduate School of Nursing Science, St. Luke’s International University, Tokyo, Japan; ^4^ Evidence Based Social Science Research Centre, School of Public Health, Lanzhou University, Lanzhou, China; ^5^ Department of Laboratory Medicine, Experimental Cancer Medicine, Karolinska Institute, Stockholm, Sweden; ^6^ Department of Respiratory, Guizhou Provincial People’s Hospital, Guiyang, China

**Keywords:** chronic obstructive pulmonary disease, long-acting muscarinic antagonist, systematic review, dose-response meta-analysis, revefenacin

## Abstract

**Background** Revefenacin (REV) is a novel once-daily long-acting muscarinic antagonist (LAMA) in the treatment of moderate to very severe chronic obstructive pulmonary disease (COPD). This systematic review incorporating a dose-response meta-analysis aimed to assess the efficacy and safety of REV.

**Methods** PubMed, Embase, Cochrane Library, China National Knowledge Infrastructure, VIP database, and Wanfang database were searched from their inception to April 2020. We included randomized controlled trials (RCTs) which evaluated the efficacy and safety of REV in COPD patients. Two reviewers independently performed study screening, data extraction, and risk of bias assessment. Outcomes consisted of the mean change in trough Forced Expiratory Volume in 1 second (FEV_1_) from baseline, adverse events (AEs), and serious adverse events (SAEs). A dose-response meta-analysis using the robust error meta-regression method was conducted. We used Grading of Recommendations, Assessment, Development and Evaluation (GRADE) approach to assess the quality of evidence.

**Results** Nine RCTs (3,121 participants) were included in this systematic review. The meta-analyses indicated that 175 μg/day REV could significantly improve the trough FEV_1_ (MD=143.67, 95%CI: 129.67 to 157.68; I^2^=96%; 809 participants; studies=4; low quality) without increasing the risk of AEs (OR=0.98, 95%CI: 0.81 to 1.18; I^2^=34%; 2,286 participants; studies=7; low quality) or SAEs (OR=0.89, 95%CI: 0.55 to 1.46; I^2^=0%; 2,318 participants; studies=7; very low quality) compared to placebo. Furthermore, the effect of REV in increasing trough FEV_1_ was dose-dependent with an effective threshold of 88 μg/day (R^2^ = 0.7017). Nevertheless, only very low-quality to low-quality evidence showed that REV at a dose of 175 μg/day was inferior to tiotropium regarding the long-term efficacy, and its safety profile was not superior to tiotropium or ipratropium.

**Conclusion** Current evidence shows that REV is a promising option for the treatment of moderate to very severe COPD. Due to most evidence graded as low quality, further studies are required to compare the efficacy, long-term safety and cost-effectiveness between REV and other LAMAs in different populations.

**Clinical Trial Registration**: [PROSPERO], identifier [CRD42020182793]

## Introduction

Chronic obstructive pulmonary disease (COPD) is a common, preventable and treatable disease that is characterized by persistent respiratory symptoms and airflow limitation due to airway and/or alveolar abnormalities (GOLD., 2021). Significant exposure to noxious particles or gases and host factors including abnormal lung development usually contribute to the pathogenesis (GOLD., 2021). Based on Burden of Obstructive Lung Disease (BOLD) and other large scale epidemiological studies, a meta-analysis estimated that the number of COPD cases was 384 million in 2010, with a global prevalence of 11.7% (95% confidence interval (CI): 8.4–15.0%) (Adeloye et al., 2015). Around 3.2 million people died from COPD each year, making it the third leading cause of death worldwide (World Health Organization, 2007; Burney et al., 2015; Global Burden of Disease Study Collaborators, 2015; Halpin et al., 2019). In the latest Global Burden of Disease (GBD) analysis, COPD entered the top 10 causes of years of life lost (YLL), increasing from the 11th position in 2007 to seventh in 2017 (GBD 2017 Causes of Death Collaborators, 2018). Another GBD study also predicted that deaths from COPD would rise to 4.4 million per year in 2040 and by then, COPD would be the fourth most important cause of YLL (Foreman et al., 2018). With the increasing exposure to risk factors (e.g., smoking) and aging of the world’s population, the prevalence of COPD is expected to rise over the next 40 years and by 2060 there may be more than 5.4 million deaths from COPD and its related conditions annually (Lopez et al., 2006; GBD 2017 Causes of Death Collaborators, 2018; World Health Organization, 2020), which will induce a substantial and elevated economic burden (Lozano et al., 2012; Vos et al., 2012). In the European Union, COPD accounted for 56% (38.6 billion Euros) of the cost on respiratory disease which took up about 6% of the total annual healthcare budget (European Respiratory Society on behalf of the Forum of International Respiratory Societies (FIRS), 2017). In the United States, the estimated direct and indirect costs of COPD were $32 billion and $20.4 billion, respectively (Guarascio et al., 2013).

In absence of conclusive evidence supporting any existing medications which can modify the long-term decline in lung function for COPD (Anthonisen et al., 1994; Burge et al., 2000; Pauwels et al., 1999; Tashkin et al., 2008; Vestbo et al., 1999), the purpose of pharmacological therapy for COPD is to ameliorate symptoms, reduce the frequency and severity of exacerbations, and improve exercise tolerance and health status. As the first-line therapy to address COPD symptoms and prevent exacerbations (GOLD., 2021), long-acting muscarinic antagonists (LAMAs) can improve the effectiveness of pulmonary rehabilitation (Casaburi et al., 2005; Kesten et al., 2008) and reduce exacerbation and related hospitalization (Karner et al., 2014; Melani A.S., 2015) by durably blocking the bronchoconstrictor effects of acetylcholine on M_3_ muscarinic receptors expressed in airway smooth muscle (Melani A.S., 2015). Revefenacin (REV), a novel once-daily LAMA for nebulization, was approved for the treatment of COPD by the United States Food and Drug Administration (FDA) in November 2018 (Highlights Of Prescribing Information, 2021). Several randomized trials (Donohue et al., 2019a; Donohue et al., 2019b; Donohue et al., 2019c; Ferguson et al., 2019; Krishna et al., 2017; Mahler et al., 2019; Quinn et al., 2018; Sethi et al., 2020; Siler et al., 2020; Theravance Biopharma, 2021a; Theravance Biopharma, 2021b) investigating the use of REV concluded that it was effective and safe in the treatment of COPD. Nevertheless, evidence has not been systematically assessed. To better understand and interpret available evidence, we conducted a systematic review incorporating a dose-response meta-analysis to evaluate the efficacy and safety of REV in patients with COPD.

## Materials and Methods

We reported our study following Preferred Reporting Items for Systematic Reviews and Meta-Analyses (PRISMA) statement (Supplementary Table S1). The study was prospectively registered on International Prospective Register of Systematic Review (PROSPERO, CRD42020182793).

### Search Strategy

PubMed, Embase, the Cochrane Central Register of Controlled Trials (CENTRAL) were searched using the search strategies detailed in Supplementary Table S2, from their inception to April 2020. ClinicalTrials.gov was also searched using the term of “Revefenacin”. The China National Knowledge Infrastructure (CNKI), VIP database, and Wanfang database were also searched with Chinese terms. We reviewed the references from relevant review articles and included studies to find additional studies.

### Eligibility Criteria

We included studies meeting the following criteria: 1) Randomized controlled trials (RCTs) published in English or Chinese; 2) participants with confirmed moderate to very severe COPD (Stage 2, three or four according to the GOLD Guidelines); 3) the intervention was REV irrespective of dosage and schedule; 4) the comparisons included placebo, tiotropium (TIO), and ipratropium (IPR); 5) studies reporting at least one of the following outcomes: the mean change from baseline in trough forced expiratory volume in 1 s (FEV_1_) as the efficacy outcome; adverse events which were subdivided into total adverse events (AEs) and serious adverse events (SAEs) by ICH GCP standards as the safety endpoints. We excluded duplicated studies or conference abstract without available raw data.

### Study Selection and Data Extraction

Two authors independently screened the titles and abstracts of all studies searched using predetermined inclusion criteria. The full texts of any potentially relevant articles were retrieved for detailed review. We resolved any disagreements by discussion. We used a pre-designed data collection form to extract data from each eligible study. The following data were extracted: 1) authors; 2) year of publication; 3) country or region where the study conducted; 4) study design and use of control; 5) number of participants in each group; 6) population characteristics (e.g., gender, age, body mass index (BMI), race, etc.); 7) outcomes and their definitions, categorical or numerical data for assessment of included outcomes; 8) Sources of funding.

### Risk of Bias Assessment

Two authors independently assessed the risk of bias of each included RCT using the checklist developed by Cochrane Collaboration (Higgins et al., 2011; Higgins et al., 2020), including random sequence generation, allocation concealment, blinding, incomplete outcome data, selective outcome reporting, and other bias. We categorized the judgement to be low, high or unclear risk of bias and created a “risk of bias summary” using the Review Manager Software (RevMan 5.3). As for crossover studies, a revised tool to assess the risk of bias in crossover trials (RoB 2) was used to assess the risk of bias (Higgins et al., 2021). Any disagreements about the risk of bias were resolved by discussion.

### Statistical Synthesis

If more than one study reported the same outcome, a pairwise meta-analysis was conducted. To compare the differences between REV and control groups, odds ratios (ORs) were used for the incidence of AEs or SAEs and mean differences (MDs) were calculated for FEV_1_, with corresponding 95% confidence intervals (CIs). We choose to use OR since a recent study have pointed out that it is better than risk ratio (RR) in clinical trials, where RR are not a portable estimator (Doi et al., 2020). As to the change from baseline in trough FEV_1_, per-protocol analyses were performed according to the data of patients who completed the trial. As to the AEs and SAEs, we conducted analyses based on the safety population which included all subjects who were randomized into the study and received at least one dose of study drug. For studies with zero-events in either of the arms, the continuity correction (add 0.5) was employed to estimate the OR and variance; for studies with zero-events in both arms, we impute OR = 1 for them while use continuity correction to estimate the variance (Xu et al., 2021). In addition, considering the unstable nature of rare events, as suggested by the guideline, we employed a sensitivity analysis by using Mantel-Haenszel risk difference (RD) estimator for the meta-analyses (Xu et al., 2021). We pooled ORs with the Mantel-Haenszel method, and MDs with the inverse variance method using RevMan 5.3, respectively. Statistical heterogeneity among studies was examined by the Chi-square test and quantified by the I^2^ statistic (Higgins et al., 2011). A fixed-effects model was applied to synthesize data when heterogeneity was not significant (I^2^<30%), while a random-effects model was used when heterogeneity was significant (I^2^>30%) and could not be explained by subgroup analyses or in terms of clinical or methodological features of the trials. We explored sources of heterogeneity based on the subgroup analyses including type of control groups and different dose of REV. The sensitivity analyses were performed by omitting the crossover studies.

The robust error meta-regression method (Xu et al., 2018) was used to summarize relationship between the dosage and response (efficacy and safety) of REV. This was achieved by treating the dosage as dependent variable (dose) while the efficacy and safety as the independent variables of study level. Under this meta-regression method, each study was regarded as a cluster within a whole population, as a solution to pool the dose-response relationship and to address the potential correlations among within-study effects. The potential dose-response relationship was fitted through a restricted cubic spline function with three random knots automatically generated. The Wald test by assuming the coefficients of non-linear terms to zero was employed to investigate whether a non-linear relationship exists (Xu et al., 2019).

### The Quality of Evidence Assessment

We used the Grading of Recommendations, Assessment, Development and Evaluation (GRADE) approach to rate the quality of evidence, which rated evidence from systematic review and meta-analysis as high, moderate, low, or very low quality, by considering risk of bias, indirectness, inconsistency, imprecision, and publication bias (Guyatt et al., 2011).

## Results

### Search Results

**FIGURE 1:**
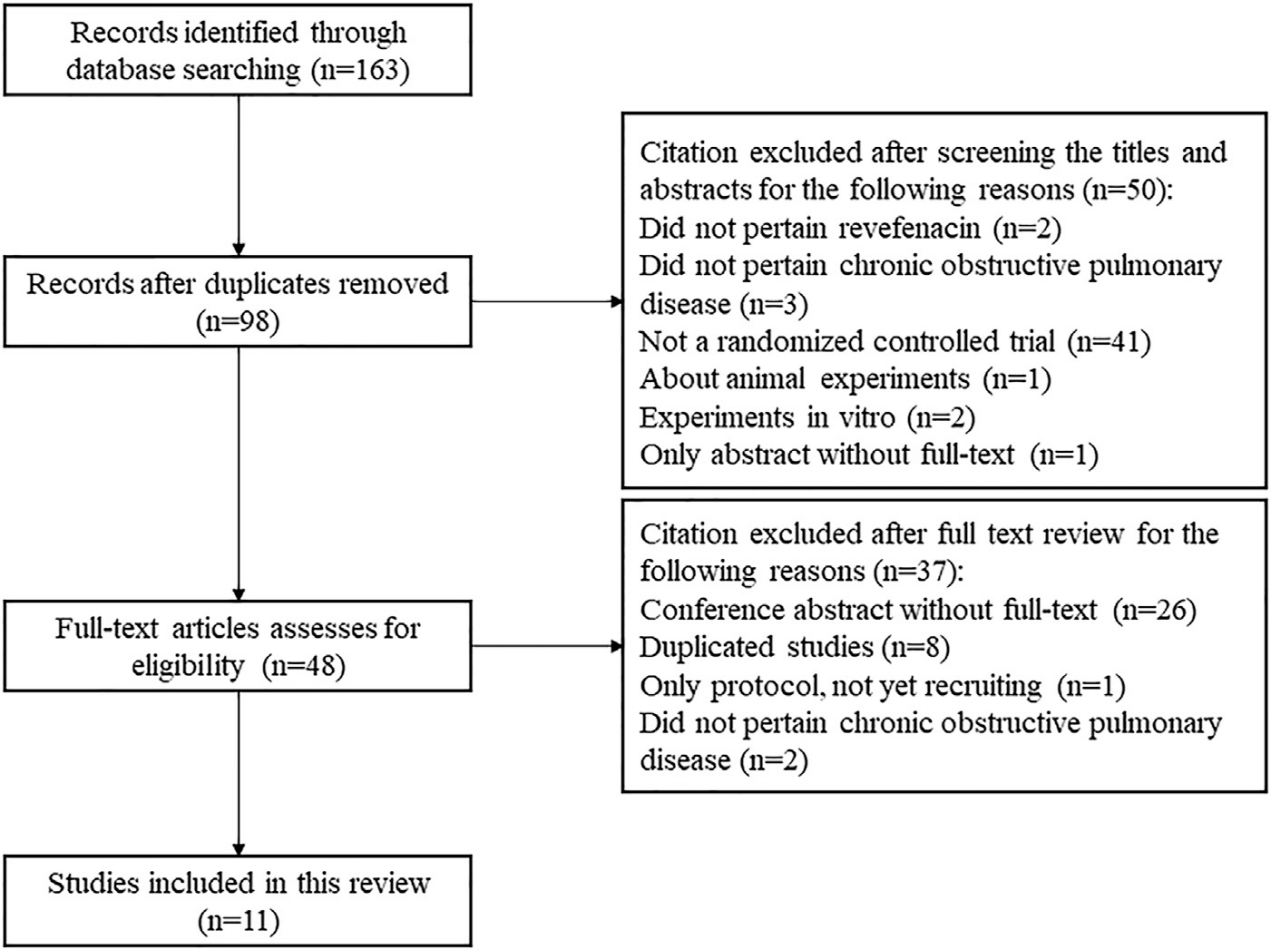
Flow diagram of study selection process for this systematic review.

A total of 163 publications were obtained from literature search and the selection process is shown in Figure 1. Eleven articles (Donohue et al., 2019a; Donohue et al., 2019b; Donohue et al., 2019c; Ferguson et al., 2019; Krishna et al., 2017; Mahler et al., 2019; Quinn et al., 2018; Sethi et al., 2020; Siler et al., 2020; Theravance Biopharma, 2021a; Theravance Biopharma, 2021b) reporting nine RCTs with 3,121 participants were included in this systematic review. As shown in Table 1, two RCTs were multicenter studies, and the other seven were single-center studies. Both parallel (n = 6) and crossover study design (n = 3) were used. The dosage of REV in intervention group ranged from 22 to 700 μg/day, and it was compared with placebo (7 RCTs, 701 participants), IPR (1 RCT, 32 participants), and TIO (2 RCTs, 460 participants). The follow-up time ranged from 1 day to 52 weeks after the first treatment. Two RCTs identified from ClinicalTrials.gov are yet to be published in full and thus the baseline characteristics of their enrolled participants were unclear. For the other seven RCTs, the mean age and mean BMI of participants were 61.4–65.1 years and 27.9–29.6 kg/m^2^, respectively, and the proportion of ICS/LABA users varied from 0 to 53.88%.

**TABLE 1:** Characteristics of included studies.

Registered ID of Trials	Study setting	Study design	Intervention vs. Control Group (n)	Age (years)	Gender: Male (%)	BMI (kg/m^2^)	Race: white (%)	Current smokers (%)	Current ICS/LABA users (%)	Baseline FEV_1_ (ml)	Follow-up time after first treatment (weeks)	Outcomes
NCT02040792	United States	Parallel	Placebo, Qd (70)	61.9 ± 8.63	178 (50.28)	27.9 ± 5.93	324 (91.52)	190 (53.67)	130 (36.72	1,283 ± 457	4	A; B; C
			REV, 44 μg, Qd (68)									
			REV, 88 μg, Qd (71)									
			REV, 175 μg, Qd (71)									
			REV, 350 μg, Qd (74)									
NCT02518139	United States	Parallel	REV, 88 μg, Qd (364)	64.4 ± 8.97	616 (58.39)	28.8 ± 6.6	977 (92.61)	489 (46.35)	560 (53.08)	1,350 ± 520	52	A; B; C
			REV, 175 μg, Qd (335)			29.1 ± 6.8				1,340 ± 490		
			TIO, 18 μg, Qd (356)			28.8 ± 6.3				1,310 ± 490		
NCT02459080	United States	Parallel	placebo, Qd (209)	64.1 ± 8.87	317 (51.21)	29.4 ± 6.6	564 (91.11)	301 (48.63)	260 (42.00)	1,400 ± 500	12	A; B; C
			REV, 88 μg, Qd (212)			29.1 ± 6.2				1,300 ± 400		
			REV, 175 μg, Qd (198)			29.6 ± 7.2				1,400 ± 500		
NCT02512510	United States	Parallel	placebo, Qd (208)	63.4 ± 8.95	302 (49.51)	29.3 ± 6.9	545 (89.34)	286 (46.88)	249 (40.82)	1,300 ± 500	12	A; B; C
			REV, 88 μg, Qd (205)			29.2 ± 7.7				1,300 ± 500		
			REV, 175 μg, Qd (197)			28.9 ± 7.0				1,300 ± 500		
NCT03095456	United States	Parallel	REV, 175 μg, Qd (102)	65.1 ± 8.13	124 (60.19)	NA	185 (89.80)	96 (46.60)	111 (53.88)	900 ± 500	4	A; B; C
			TIO, 18 μg, Qd (104)									
NCT03573817	United States	Parallel	REV, 175 µg, Qd	63.7 ± 8.56	69 (56.56)	29.17 ± 6.475	116 (95.08)	69 (56.56)	28 (22.95)	1,340 ± 480 1,340 ± 500	6	B; C
			+ FOR, 20 µg, Bid (63)									
			Placebo, Qd									
			+ FOR, 20 µg, Bid (59)									
NCT01704404	United KingdomNorthern Ireland New Zealand	Crossover	REV, 22 μg, Qd (40)	63.9 (45–75)	33 (55.93	28.8 ± 5.92	59 (100)	NA	0 (0)	1,600 ± 500	1	A; B; C
			REV, 44 μg, Qd (39)									
			REV, 88 μg, Qd (39)									
			REV, 175 μg, Qd (39)									
			REV, 350 μg, Qd (39)									
			REV, 700 μg, Qd (40)									
			Placebo, Qd (59)									
NCT02109172	United States	Crossover	REV, 44 μg, Bid (64)	40–65: n = 39 y≥ 65: n = 25	37 (57.81)	NA	NA	NA	NA	NA	1	B; C
			REV, 175 μg, Qd (64)									
			Placebo, Qd (64)									
NCT03064113 Or U1111-1,120–8,290	South Africa New Zealand	Crossover	Placebo, Qd (32)	18–65: n = 22 y≥ 65: n = 10	22 (68.75%)	27.72 ± 8.0	28 (87.5)	NA	NA	1900 ± 500	1 day	B; C
			REV, 350 μg, Qd (32)									
			REV, 700 μg, Qd (32)									
			IPR, 500 μg, Qd (32)									

n: sample size; BMI: body mass index; FEV_1_: Forced Expiratory Volume in 1 s; REV: revefenacin; TIO: tiotropium; FOR: formoterol; IPR: ipratropium; NA: not applicable; A: change from baseline in trough FEV_1_; B: total adverse events (AEs); C: serious adverse events (SAEs).

### Quality of Included Studies

**FIGURE 2:**
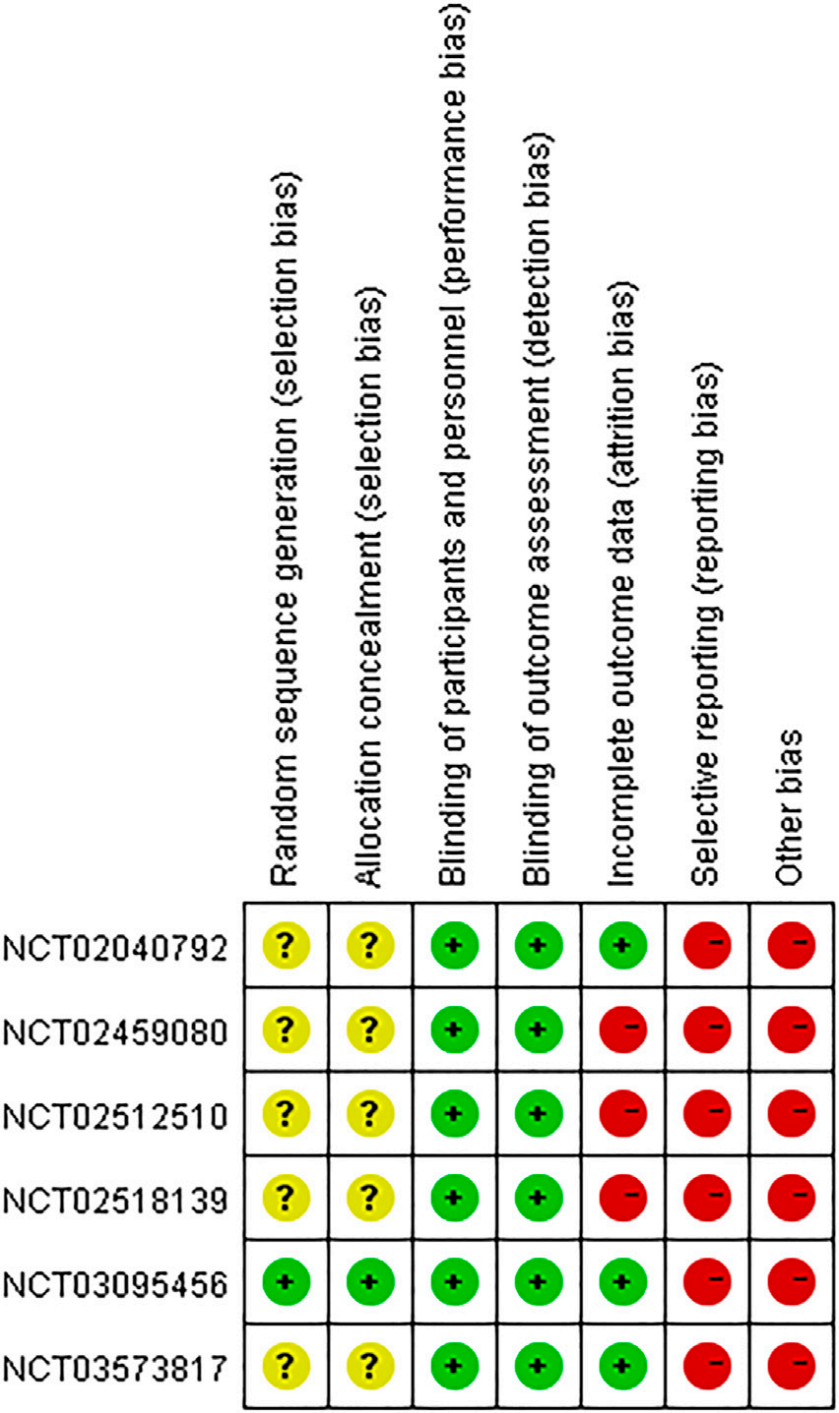
Risk of bias summary of parallel studies.

As shown in Figure 2, one study (NCT03095456) had low risk of selection bias for clearly describing the methods (centralized randomization) of randomization and allocation concealment, while the others were unclear because the information about selection participants was not reported. Triple (participant, care provider, and investigator) and quadruple (participant, care provider, investigator, and outcome assessor) blinding methods were applied in three RCTs (NCT02040792, NCT02459080, NCT02512510) and three RCTs (NCT02040792, NCT03095456, NCT03573817), respectively, therefore all the included studies had low risk of performance bias and detection bias. Four studies (NCT02040792, NCT03095456, NCT03573817, NCT02109172) had low risk of attribution bias, as there was no loss of follow-up or missing data was appropriately addressed (e.g., applying ITT analysis which could underestimate the efficacy of the intervention). Nevertheless, other three studies (NCT02518139, NCT02459080, NCT02512510) had high risk of attribution bias due to high loss of follow-up (>15%). Although all the studies mentioned registration information and had an available protocol, data from some outcomes of interest (i.e., AEs, SAEs, FEV_1_) in six studies (NCT02040792, NCT02518139, NCT02459080, NCT02512510, NCT03095456, NCT03573817) were inconsistent with the information on ClinicalTrial.gov. Therefore, the reporting bias risk of these studies was high. Since Theravance Biopharma, Inc. supported all the studies and their employees participated in the executing and writing process of six studies (NCT02040792, NCT02518139, NCT02459080, NCT02512510, NCT03095456, NCT03573817), the risk of bias caused by conflict of interest was high. Due to the limited number of the included studies for the same outcome, publication bias investigation was not performed. As to the three crossover studies (NCT01704404, NCT02109172, and NCT03064113), the overall risk of bias was assessed as “some concerns” (Table 2).

**TABLE 2:** Risk of bias summary of cross-over studies.

Registered ID of Trials	Risk of bias arising from the randomization process	Risk of bias arising from period and carryover effects	Risk of bias due to deviations from the intended interventions (effect of assignment to intervention)	Risk of bias due to deviations from the intended interventions (effect of adhering to intervention)	Risk of bias due to missing outcome data	Risk of bias in measurement of the outcome	Risk of bias in selection of the reported result	Overall risk of bias
NCT01704404	Low risk	Low risk	Some concerns	Low risk	Low risk	Low risk	Some concerns	Some concerns
NCT02109172	Some concerns	Low risk	Some concerns	Low risk	Low risk	Low risk	Low risk	Some concerns
NCT03064113	Some concerns	Some concerns	Some concerns	Low risk	Low risk	Low risk	Low risk	Some concerns

### Results From the Meta-analysis

#### The Change From Baseline in Trough FEV_1_

**FIGURE 3:**
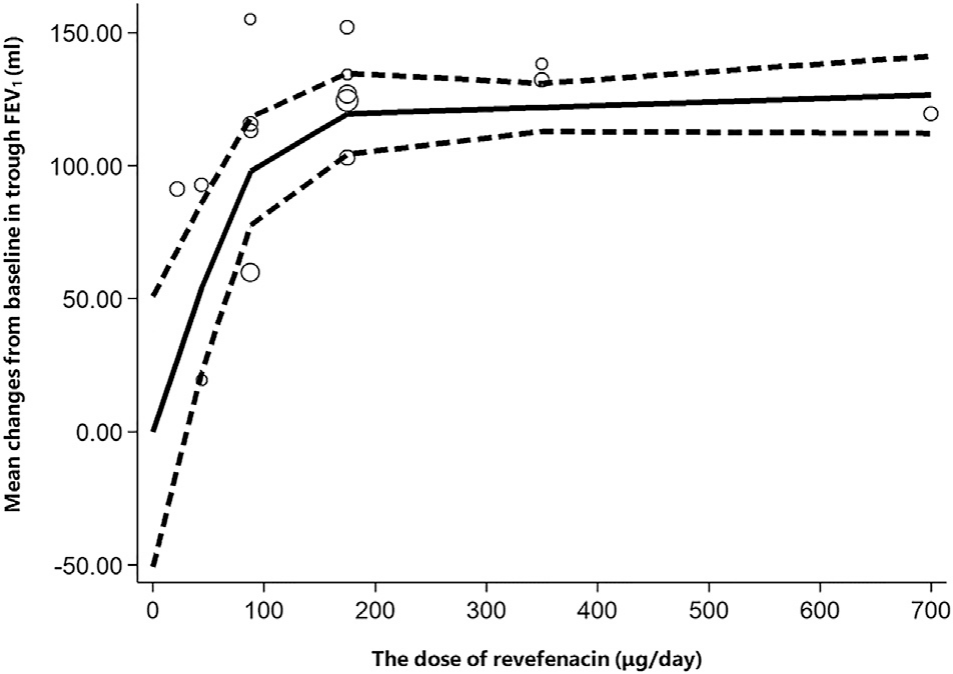
Increase in dose (μg/day) of revefenacin and change from baseline in trough FEV1 (ml). The solid line is the nonlinear prediction of the mean change from baseline in trough FEV_1_ and the dotted lines indicate the 95% confidence interval. The threshold is 88 μg/day. FEV_1_: Forced Expiratory Volume in 1 s.

Six trials involving 2,093 participants reported the change from baseline in trough FEV_1_. Among them, four trials (NCT02040792, NCT02459080, NCT02512510, and NCT01704404) compared REV with placebo at different doses, one trial (NCT02518139) compared REV with TIO at different follow-up time (4-weeks, 13-weeks, 26-weeks, 39-weeks, and 52-weeks), whereas the rest one (NCT03095456) made plain comparison between REV and TIO. In subgroup analyses, we found that both dose and therapeutic course of REV contribute to the heterogeneity, so the results were presented according to the control group, the dose and the therapeutic course (Table 3). In contrast to placebo, all different doses of REV could significantly improve the trough FEV_1_. Yet this effect would be weakened with the longer course of treatments. Despite that trials NCT02459080 and NCT02512510 reported the change from baseline in trough FEV_1_ for 88 μg/day REV vs. placebo at 12-weeks, the heterogeneity between the two trials was significantly high (I^2^ = 100%). Therefore, we described their respective results rather than the pooling results. In the dose-response meta-analysis, there was a potential non-linear association (*R* ^2^ = 0.7017) of the REV dose with the change from baseline in trough FEV_1_ (Figure 3). The predicted dose-specific mean changes from baseline in trough FEV_1_ were 27.43 (95%CI: 13.55–68.41) ml at a dose of 22 μg/day, 54.41 (95%CI: 22.50–86.31) ml at a dose of 44 μg/day, 97.96 (95%CI: 77.72–118.21) ml at a dose of 88 μg/day, 119.47 (95%CI: 104.21–134.74) ml at a dose of 175 μg/day, 121.86 (95%CI: 112.79–130.92) ml at a dose of 350 μg/day, and 126.63 (95%CI: 112.13–141.12) ml at a dose of 700 μg/day. Interestingly, 88 μg/day seemed to be a threshold dose above which the change from baseline in trough FEV1 began to slow down (Figure 3). Patients who received 175 μg/day REV experienced improvement of trough FEV_1_ on average of 143.67 ml higher than those who received placebo (MD = 143.67, 95%CI: 129.67 to 157.68; I^2^ = 96%; 809 participants; studies = 4; low quality; Table 4). Patients treated with 175 μg/day REV gained increment of trough FEV_1_ on average of 13.51 ml higher than TIO at 4 weeks (MD = 13.51, 95%CI: 8.32 to 18.69; I^2^ = 66%; 791 participants; studies = 2; very low quality; Table 4), but this effect was reversed at 52 weeks (MD = -39.2, 95%CI: 41.82 to 36.58; 433 participants; study = 1; low quality; Table 4). The sensitivity analyses showed that the results including crossover studies were consistent with those omitting crossover studies (Supplementary Table S3).

**TABLE 3:** The results of the pairwise meta-analysis of change from baseline in trough FEV_1_.

Group	Follow-up time	*N*	*n*	Heterogeneity	Model	*MDs(ml)*	95%*CIs*	*P*
REV 22 vs PLA	1 week	1	37 vs. 56	*NA*	*NA*	53.40	(45.79, 61.01)	<0.00001
REV 44 vs PLA	1 week	1	32 vs. 56	*NA*	*NA*	55.00	(46.70, 63.30)	<0.00001
	4 weeks	1	60 vs. 55	*NA*	*NA*	51.80	(42.59, 61.01)	<0.00001
REV 88 vs PLA	1 week	1	35 vs 56	*NA*	*NA*	75.30	(67.45, 83.15)	<0.00001
	4 weeks	1	63 vs. 55	*NA*	*NA*	187.40	(178.35, 196.45)	<0.00001
	12 weeks	1	161 vs. 146	*NA*	*NA*	79.22	(75.72, 82.72)	<0.00001
	12 weeks	1	152 vs. 150	*NA*	*NA*	160.50	(156.27, 164.73)	<0.00001
REV 175 vs PLA	1 week	1	33 vs. 56	*NA*	*NA*	114.10	(105.96, 122.24)	<0.00001
	4 weeks	1	59 vs. 55	*NA*	*NA*	166.60	(157.33, 175.87)	<0.00001
	12 weeks	2	310 vs. 296	*I* ^*2*^ = 0%, *p* = 0.58	Fixed	146.91	(144.20, 149.63)	<0.00001
REV 350 vs PLA	1 week	1	38 vs. 56	*NA*	*NA*	94.40	(86.90, 101.90)	<0.00001
	4 weeks	1	63 vs. 55	*NA*	*NA*	170.60	(161.59, 179.61)	<0.00001
REV 700 vs PLA	1 week	1	35 vs. 56	*NA*	*NA*	81.60	(73.75, 89.45)	<0.00001
REV 88 vs TIO	4 weeks	1	317 vs. 330	*NA*	*NA*	-29.00	(−30.82, −27.18)	<0.00001
	13 weeks	1	287 vs. 307	*NA*	*NA*	-16.00	(−17.96, −14.04)	<0.00001
	26 weeks	1	239 vs. 283	*NA*	*NA*	-14.80	(−16.99, −12.61)	<0.00001
	39 weeks	1	223 vs. 265	*NA*	*NA*	-10.20	(−12.51, −7.89)	<0.00001
	52 weeks	1	212 vs. 248	*NA*	*NA*	-42.70	(−45.12, −40.28)	<0.00001
REV 175 vs TIO	4 weeks	2	371 vs. 420	*I* ^*2*^ = 66%, *p* = 0.08	Random	13.51	(8.32, 18.69)	<0.00001
	13 weeks	1	243 vs. 307	*NA*	*NA*	2.70	(0.55, 4.85)	<0.00001
	26 weeks	1	210 vs. 283	*NA*	*NA*	15.40	(13.03, 17.77)	<0.00001
	39 weeks	1	189 vs. 265	*NA*	*NA*	-8.30	(−10.85, −5.75)	<0.00001
	52 weeks	1	185 vs. 248	*NA*	*NA*	-39.20	(−41.82, −36.58)	<0.00001

FEV_1_: Forced Expiratory Volume in 1 s; N: the number of included trials; n: the number of participants; MDs: mean differences; 95%CIs: 95% confidence intervals; REV 22: revefenacin 22 μg/day; REV 44: revefenacin 44 μg/day; REV 88: revefenacin 88 μg/day; REV 175: revefenacin 175 μg/day; REV 350: revefenacin 350 μg/day; REV 700: revefenacin 700 μg/day; PLA: placebo; TIO: tiotropium; Fixed: fixed-effects model; Random: random-effects model; NA: not applicable.

**TABLE 4:** GRADE summary of findings for intervention versus controls in patients with chronic obstructive pulmonary disease (COPD).

Patient or population	Settings	Intervention	Comparison	Outcomes (timeframe)	Relative effect (95%CI)	No. of participants	Absolute effect estimate (95%CI)	Quality of evidence	Comments
Individuals with COPD	Outpatient	Revefenacin 175 μg/day	Placebo	Change from baseline in trough FEV_1_ (ml) (From 10 week to 12 weeks)	NA	809 patients in 4 RCTs	143.67 higher (129.67 higher to 157.68 higher)	Low^a, b, c^	Revefenacin 175 μg/day might improve lung function compared to placebo.
Individuals with COPD	Outpatient	Revefenacin 175 μg/day	Tiotropium 18 μg/day	Change from baseline in trough FEV_1_ (ml) (At 4 weeks)	NA	791 patients in 2 RCTs	13.51 higher (8.32 higher to 18.69 higher)	Very low^a, d^	Revefenacin 175 μg/day might improve lung function compared to tiotropium in the short term.
Individuals with COPD	Outpatient	Revefenacin 175 μg/day	Tiotropium 18 μg/day	Change from baseline in trough FEV_1_ (ml) (At 52 weeks)	NA	433 patients in one RCT	39.2 lower (41.82 lower to 36.58 lower)	Low^a,d^	Revefenacin 175 μg/day might not improve lung function compared to tiotropium in the long term.
Individuals with COPD	Outpatient	Revefenacin 22–700 μg/day	Placebo	Any adverse events (From 1 day to 12 weeks)	Odds ratio: 0.98 (0.81–1.18)	2,286 patients in 7 RCTs	5 fewer (51 fewer to 41 more)	Low^a^	Revefenacin might not increase the risk of any adverse events compared to placebo.
Individuals with COPD	Outpatient	Revefenacin 88–175 μg/day	Tiotropium 18 μg/day	Any adverse events (From 4 to 52 weeks)	Odds ratio: 0.44 (0.12–1.60)	1,262 patients in 2 RCTs	197 fewer (477 fewer to 92 more)	Very low^a,e,f^	Revefenacin might not increase the risk of any adverse events compared to tiotropium.
Individuals with COPD	Outpatient	Revefenacin 350–700 μg/day	Ipratropium 500 μg/day	Any adverse events (At 1 day)	Odds ratio: 0.66 (0.23–1.94)	96 patients in one RCT	63 fewer (158 fewer to 133 more)	Very Low^a,f,g^	Revefenacin might not increase the risk of any adverse events compared to ipratropium.
Individuals with COPD	Outpatient	Revefenacin 22–700 μg/day	Placebo	Serious adverse events (From 1 day to 12 weeks)	Odds ratio: 0.89 (0.55–1.46)	2,318 patients in 7 RCTs	4 fewer (14 fewer to 14 more)	Very low^a,f^	Revefenacin might not increase the risk of serious adverse events compared to placebo.
Individuals with COPD	Outpatient	Revefenacin 88–175 μg/day	Tiotropium 18 μg/day	Serious adverse events (From 4 to 52 weeks)	Odds ratio: 0.86 (0.61–1.21)	1,262 patients in 2 RCTs	16 fewer (46 fewer to 23 more)	Low^a^	Revefenacin might not increase the risk of serious adverse events compared to tiotropium.
Individuals with COPD	Outpatient	Revefenacin 350–700 μg/day	Ipratropium 500 μg/day	Serious adverse events (At 1 day)	Odds ratio: 1.00 (0.13–7.43)	96 patients in one RCT	0	Low^g,h^	Revefenacin might not increase the risk of serious adverse events compared to ipratropium.

CI: confidence interval; FEV_1_: Forced Expiratory Volume in 1 s; RCT: randomized controlled trial; PLA: placebo; ^a^: very serious risk of bias (unclear selection bias, high risk of attribution, reporting, and other bias); ^b^: very considerable inconsistence (I^2^ = 96%, high heterogeneity caused by different timeframe and disparate results across studies); ^c^: upgraded because all plausible confounding would reduce demonstrated effect and the dose-response gradient was strong; ^d^: considerable heterogeneity (I^2^ = 66%); ^e^: very considerable inconsistence (I^2^ = 91%, high heterogeneity caused by different timeframe and non-overlapping 95% CIs); ^f^: wide 95% CI with a lower limit <0.75 and an upper limit >1.25; ^g^: serious risk of bias (unclear selection and other bias); ^h^: small sample size.

#### The Incidence of Any Adverse Events

**FIGURE 4:**
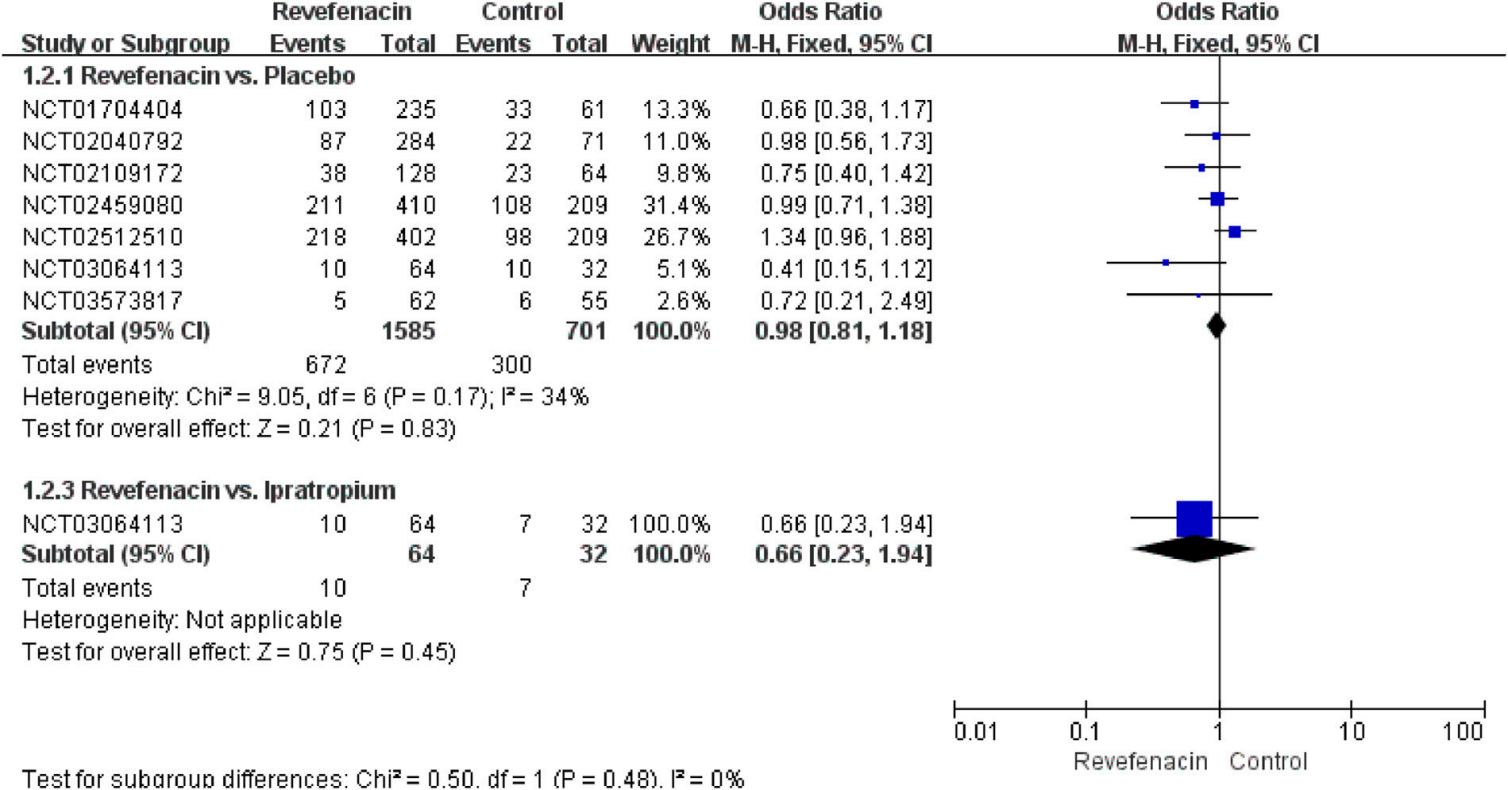
Total adverse events for revefenacin in patients with chronic obstructive pulmonary disease.

The AEs were reported in all trials including 3,121 participants. As presented in Table 5, most AEs were mild, transient, and reversible. A limited association (*R* ^2^ = 0.1787) of the REV dose with the total AEs incidence was present (Supplementary Figure S1). The predicted dose-specific RRs of the REV dose were 1.03 (95%CI: 1.00–1.07) at a dose of 22 μg/day, 1.02 (95%CI: 0.99–1.06) ml at a dose of 44 μg/day, 1.00 (95%CI: 0.97–1.04) ml at a dose of 88 μg/day, 0.96 (95%CI: 0.92–1.01) ml at a dose of 175 μg/day, 0.89 (95%CI: 0.81–0.97) ml at a dose of 350 μg/day, and 0.76 (95%CI: 0.64–0.90) ml at a dose of 700 μg/day. On average, the decrease in total AEs was 0.05% (RR = 0.9995, 95%CI: 0.9992–0.9998; *p* = 0.009) between 0 and the maximum dose. Furthermore, tests of interaction showed no evidence of different therapeutic course subgroup effect for total AEs in comparison of REV vs. PLA (Supplementary Figure S2). Notably, the incidence of total AEs in REV group was significantly lower than that in TIO group at 4 weeks (OR = 0.22, 95%CI: 0.11–0.45, *p* < 0.0001), while the difference became not significant at 52 weeks (OR = 0.82, 95%CI: 0.61–1.10, *p* = 0.19). Patients who received REV were the equivalent likely to undergo total AEs as placebo patients (OR = 0.98, 95%CI: 0.81 to 1.18; I^2^ = 34%; 2,286 participants; studies = 7; low quality; Figure 4, Table 3), TIO patients (OR = 0.44, 95%CI: 0.12 to 1.60; I^2^ = 91%; 1,262 participants; studies = 2; very low quality; Supplementary Figure S3, Table 4), or IPR patients (OR = 0.66, 95%CI: 0.23 to 1.94; 96 participants; study = 1; very low quality; Figure 4, Table 4). The sensitivity analyses showed that the results including crossover studies were consistent with those omitting crossover studies (Supplementary Figure S4).

**TABLE 5:** The incidence of non-serious adverse events for revefenacin.

Non-serious adverse events	Events	Total	Incidence (%)
**Infections and infestations**	—	—	—
Nasopharyngitis	83	2,450	3.39
Upper respiratory tract infection	75	2,450	3.06
Bronchitis	34	2,450	1.39
Urinary tract infection	31	2,450	1.27
Sinusitis	31	2,450	1.27
Tooth infection	1	2,450	0.04
Viral infection	1	2,450	0.04
Acute sinusitis	1	2,450	0.04
Ear Infection	1	2,450	0.04
Furuncle	1	2,450	0.04
**Investigations**	—	—	—
Electrocardiogram T wave peaked	3	2,450	0.12
**Metabolism and nutrition disorders**	—	—	—
Gout	2	2,450	0.08
**Nervous system disorders**	—	—	—
Headache	103	2,450	4.20
Dizziness	1	2,450	0.04
Tremor	1	2,450	0.04
**Respiratory, thoracic and mediastinal disorders**	—	—	—
Chronic obstructive pulmonary disease exacerbation	273	2,450	11.14
Cough	95	2,450	3.88
Dyspnea	90	2,450	3.67
Pneumonia	21	2,450	0.86
Dysphonia	1	2,450	0.04
Chest Discomfort	2	2,450	0.08
Rhinorrhea	3	2,450	0.12
Oropharyngeal pain	6	2,450	0.24
Rhonchi	1	2,450	0.04
Sputum increased	1	2,450	0.04
**Gastrointestinal disorders**	—	—	—
Diarrhea	27	2,450	1.10
Gastroesophageal reflux disease	16	2,450	0.65
Nausea	16	2,450	0.65
Dry mouth	3	2,450	0.12
Oral discomfort	1	2,450	0.04
Inguinal hernia	1	2,450	0.04
Vomiting	1	2,450	0.04
**General disorders**	—	2,450	—
Fatigue	4	2,450	0.16
Oedema	2	2,450	0.08
**Injury, poisoning and procedural complications**	—	—	—
Contusion	6	2,450	0.24
Muscle contusion	1	2,450	0.04
Eye swelling	1	2,450	0.04
Eye contusion	1	2,450	0.04
Procedural pain	1	2,450	0.04
**Musculoskeletal and connective tissue disorders**	—	—	—
Back pain	37	2,450	1.51
Arthralgia	15	2,450	0.61
Pain in extremity	1	2,450	0.04
Muscle spasms	1	2,450	0.04
Musculoskeletal pain	1	2,450	0.04
Neck pain	1	2,450	0.04
**Neoplasms benign, malignant and unspecified (incl cysts and polyps)**	—	—	—
Basal cell carcinoma	1	2,450	0.04
**Skin and subcutaneous tissue disorders**	—	2,450	—
Rash	6	2,450	0.24
Dermatitis contact	2	2,450	0.08
Skin lesion	1	2,450	0.04
**Vascular disorders**		2,450	
Hypertension	27	2,450	1.10
Hematoma	2	2,450	0.08
Blood pressure increased	1	2,450	0.04
Hypotension	1	2,450	0.04
Coronary artery insufficiency	1	2,450	0.04
**Psychiatric disorders**	—	—	—
Insomnia	1	2,450	0.04

#### The Incidence of SAEs

**FIGURE 5:**
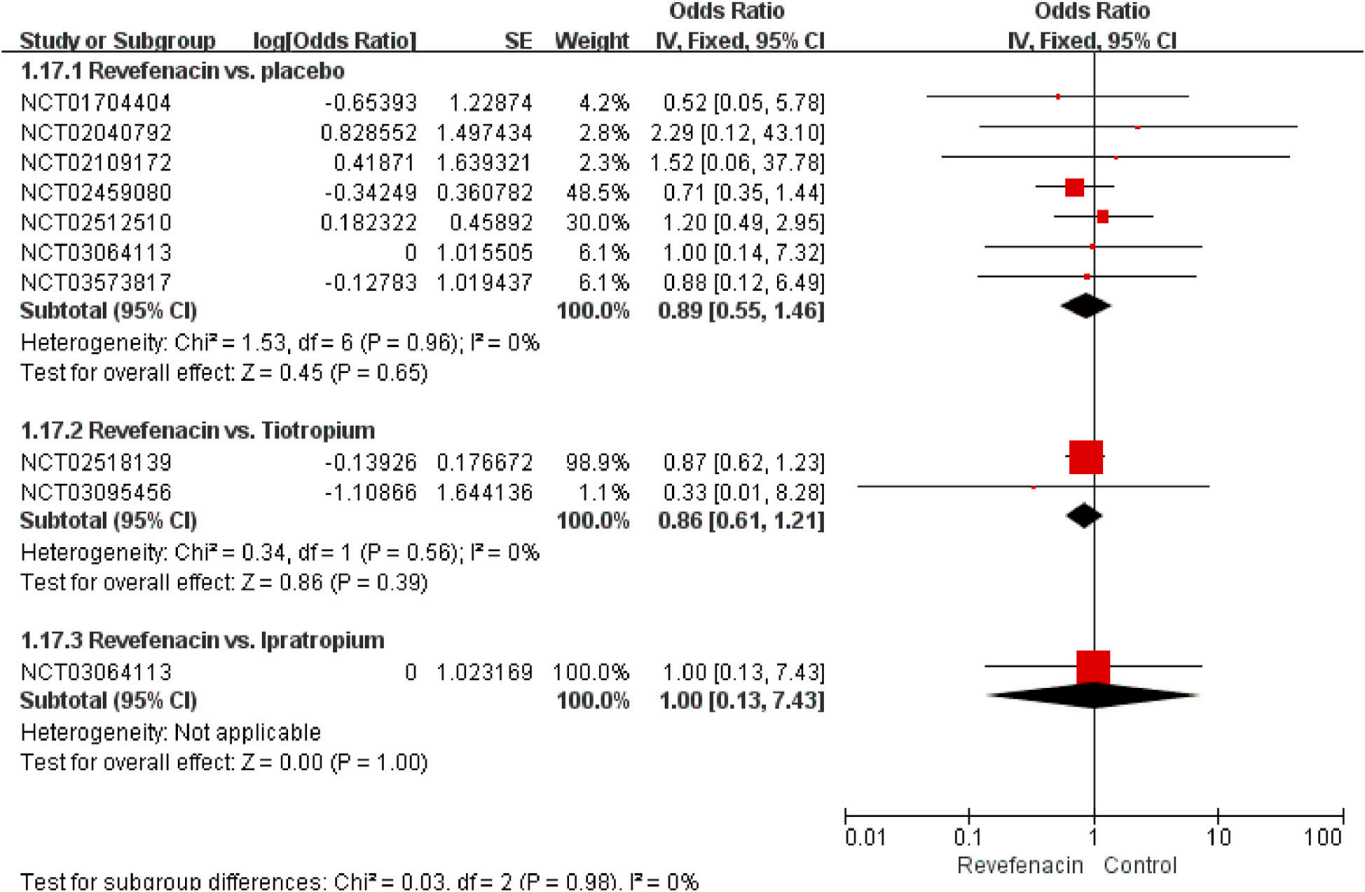
Serious adverse events for revefenacin in patients with chronic obstructive pulmonary disease.

All the nine trials reported 200 SAEs, and the most common SAEs was COPD worsening or exacerbation (1.39%, Table 6). A weak association (*R* ^2^ = 0.1325) of the REV dose with the SAEs incidence existed (Supplementary Figure S5). The predicted dose-specific RRs of the REV dose were 0.99 (95%CI: 0.95–1.04) at a dose of 22 μg/day, 0.97 (95%CI: 0.93–1.02) ml at a dose of 44 μg/day, 0.94 (95%CI: 0.88–0.99) ml at a dose of 88 μg/day, 0.86 (95%CI: 0.78–0.96) ml at a dose of 175 μg/day, 0.74 (95%CI: 0.60–0.90) ml at a dose of 350 μg/day, and 0.54 (95%CI: 0.36–0.81) ml at a dose of 700 μg/day. The average decrement in risk of SAEs between 0 and the maximum dose was 0.1% (RR = 0.9990, 95%CI: 0.9984–0.9998; *p* = 0.020). Yet we found no evidence of different therapeutic course effect for this outcome in comparison of REV vs. PLA (Supplementary Figure S6). Patients treated with REV were the similar likely to experience SAEs as placebo patients (OR = 0.89, 95%CI: 0.55 to 1.46; I^2^ = 0%; 2,318 participants; studies = 7; very low quality; Figure 5, Table 4), TIO patients (OR = 0.86, 95%CI: 0.61 to 1.21; I^2^ = 0%; 1,262 participants; studies = 2; low quality; Figure 5, Table 4), or IPR patients (OR = 1.00, 95%CI: 0.13 to 7.43; 96 participants; study = 1; low quality; Figure 5, Table 4). These results were consistent with the sensitivity analyses by using Mantel-Haenszel RD (Supplementary Figure S7). The sensitivity analyses showed that the results including crossover studies were consistent with those omitting crossover studies (Supplementary Figure S8).

**TABLE 6:** The incidence of serious adverse events for revefenacin.

Serious adverse events	Events	Total	Incidence (%)
**Cardiac disorders**	—	—	—
Myocardial infarction	6	2,450	0.24
Acute myocardial infarction	6	2,450	0.24
Angina unstable	3	2,450	0.12
Coronary artery occlusion	2	2,450	0.08
Cardiac failure congestive	2	2,450	0.08
Coronary Artery Insufficiency	1	2,450	0.04
Atrial fibrillation	1	2,450	0.04
Silent myocardial infarction	1	2,450	0.04
Acute coronary syndrome	1	2,450	0.04
Cardiac arrest	1	2,450	0.04
Angina pectoris	1	2,450	0.04
Bradycardia	1	2,450	0.04
Coronary artery disease	1	2,450	0.04
Tachycardia	1	2,450	0.04
**Gastrointestinal disorders**	—	—	—
Small intestinal obstruction	3	2,450	0.12
Upper gastrointestinal hemorrhage	3	2,450	0.12
Colitis	2	2,450	0.08
Diverticulum intestinal hemorrhagic	2	2,450	0.08
Pancreatitis acute	2	2,450	0.08
Intestinal obstruction	1	2,450	0.04
Gastric volvulus	1	2,450	0.04
Abdominal pain	1	2,450	0.04
Gastrointestinal hemorrhage	1	2,450	0.04
Nausea	1	2,450	0.04
Pancreatic mass	1	2,450	0.04
Rectal hemorrhage	1	2,450	0.04
**Vascular disorders**			
Hypertension	1	2,450	0.04
Hypotension	1	2,450	0.04
Accelerated hypertension	1	2,450	0.04
Aortic aneurysm	1	2,450	0.04
Circulatory collapse	1	2,450	0.04
Peripheral arterial occlusive disease	1	2,450	0.04
**Endocrine disorders**			
Goitre	1	2,450	0.04
**General disorders**	—	—	—
Non-cardiac chest pain	5	2,450	0.20
Chest pain	5	2,450	0.20
Impaired healing	1	2,450	0.04
Cardiac death	1	2,450	0.04
Systemic inflammatory response syndrome	1	2,450	0.04
**Hepatobiliary disorders**			
Jaundice	1	2,450	0.04
**Infections and infestations**	—	—	—
Pneumonia	12	2,450	0.49
Cellulitis	4	2,450	0.16
Bronchitis	3	2,450	0.12
Appendicitis	2	2,450	0.08
Bronchitis bacterial	1	2,450	0.04
Pneumonia para-influenzae viral	1	2,450	0.04
Diverticulitis	1	2,450	0.04
Pneumonia bacterial	1	2,450	0.04
Abscess neck	1	2,450	0.04
Infected skin ulcer	1	2,450	0.04
Ludwig angina	1	2,450	0.04
Osteomyelitis	1	2,450	0.04
Post procedural infection	1	2,450	0.04
Sepsis	1	2,450	0.04
**Injury, poisoning and procedural complications**	—	—	—
Femur fracture	1	2,450	0.04
Hip fracture	1	2,450	0.04
Lower limb fracture	1	2,450	0.04
Multiple fractures	1	2,450	0.04
Road traffic accident	1	2,450	0.04
Upper limb fracture	1	2,450	0.04
**Musculoskeletal and connective tissue disorders**	—	—	—
Osteoarthritis	4	2,450	0.16
Cervical spinal stenosis	2	2,450	0.08
Musculoskeletal chest pain	2	2,450	0.08
Rheumatoid arthritis	1	2,450	0.04
Muscular weakness	1	2,450	0.04
Spinal column stenosis	1	2,450	0.04
**Neoplasms benign, malignant and unspecified (incl cysts and polyps)**	—	—	—
Lung neoplasm malignant	2	2,450	0.08
Small cell lung cancer	2	2,450	0.08
Colon cancer	2	2,450	0.08
Lung adenocarcinoma	2	2,450	0.08
Uterine leiomyoma	1	2,450	0.04
Brain cancer metastatic	1	2,450	0.04
Colon cancer stage 0	1	2,450	0.04
Hepatic cancer	1	2,450	0.04
Lung carcinoma cell type unspecified stage IV	1	2,450	0.04
Ovarian cancer	1	2,450	0.04
Prostate cancer	1	2,450	0.04
Pancreatic carcinoma	1	2,450	0.04
Squamous cell carcinoma	1	2,450	0.04
Breast cancer	1	2,450	0.04
**Nervous system disorders**	—	—	—
Transient ischemic attack	1	2,450	0.04
Migraine	1	2,450	0.04
Carotid artery stenosis	1	2,450	0.04
Depressed level of consciousness	1	2,450	0.04
Syncope	1	2,450	0.04
**Renal and urinary disorders**	—	—	—
Renal artery stenosis	1	2,450	0.04
**Reproductive system and breast disorders**	—	—	—
Benign prostatic hyperplasia	1	2,450	0.04
**Respiratory, thoracic and mediastinal disorders**	—	—	—
Chronic obstructive pulmonary disease	34	2,450	1.39
Acute respiratory failure	8	2,450	0.33
Dyspnea	2	2,450	0.08
Pulmonary embolism	2	2,450	0.08
Respiratory failure	2	2,450	0.08
Bronchiectasis	1	2,450	0.04
Pleural effusion	1	2,450	0.04
Pulmonary granuloma	1	2,450	0.04
Pulmonary mass	1	2,450	0.04
Pneumothorax	1	2,450	0.04
Hypoxia	1	2,450	0.04
**Skin and subcutaneous tissue disorders**	—	—	—
Hyperhidrosis	1	2,450	0.04
Subcutaneous emphysema	1	2,450	0.04
**Psychiatric disorders**	—	—	—
Panic attack	1	2,450	0.04
Bipolar disorder	1	2,450	0.04
**Metabolism and nutrition disorders**	—	—	—
Lactic acidosis	1	2,450	0.04

## Discussion

This systematic review summarized the evidence of efficacy and safety of REV in patients with moderate to very severe COPD and used a novel meta-analysis method to account for the dose-response relationship of the trough FEV_1_, AEs, and SAEs with REV dose. Low-quality evidence suggests that, compared to placebo, 175 μg/day REV might improve the lung function (increment of trough FEV_1_ on average of 143.67 ml higher than placebo) without elevating the risk of AEs or SAEs. However, only very low-quality to low-quality evidence demonstrates that the safety profile of REV at a dose of 175 μg/day is similar to TIO and IPR but its long-term efficacy is inferior to TIO (decrease of trough FEV_1_ on average of 39.2 ml lower than TIO). The effect of REV in increasing trough FEV_1_ was correlated to the dose with a threshold value of 88 μg/day. Notably, the efficacy of REV would be weakened with the extension of therapeutical course.

Despite the serious risk of bias and inconsistence, the confidence rating of evidence regarding the efficacy of REV vs. placebo might be enhanced by the dose-response gradient which was consistent with the results *in vitro* (Pulido-Rios et al., 2013). The novel robust error meta-regression method had some merits of reducing the probability of type I error caused by repeated analyses, so it was utilized to investigate the dose-response relationship. This relationship was non-linear and included three phases based on REV dose: 0–88 μg/day, 88–175 μg/day, and 175–700 μg/day. The change from baseline in trough FEV_1_ dramatically escalated with the increasing dose of REV from 0 to 88 μg/day. Thereafter, the growth rate started to slow down and achieved a plateau phase when the dose exceeded 175 μg/day due to a ceiling effect. Our finding is coincided with current suggestion where 88 and 175 ug/day REV are considered as appropriate doses for investigating longer-term safety and efficacy of REV (Krishna et al., 2017). To explore the heterogeneity of trough FEV_1_ regarding 88 μg/day of REV vs. placebo at 12-weeks, we compared the baseline of participants in trial NCT02512510 with that in trial NCT02459080. Unfortunately, there was no significant difference in baseline characteristics. Hence, one possible reason for explaining the heterogeneity is that a dose of 88 μg/day was the threshold of the dose-response curve and some patients in the study might not receive the full benefits of the treatment, suggesting that a higher dose would be more optimal for all participants. Different from efficacy, there was no significant dose-response relationship between dose and the incidence of AEs or SAEs and the safety profile of REV was comparable to placebo. In addition, a previous study also reported that concurrent long-acting β-agonists (LABA) would slightly raise the incidence of AEs for patients receiving REV at a dose of 88 μg/day rather than those receiving REV at a dose of 175 μg/day (Donohue et al., 2019c). Thereby 175 μg/day has been approved as a standard dose by the United States FDA (Highlights Of Prescribing Information, 2021).

The effect of REV at a dose of 175 μg/day in improving the trough FEV_1_ was superior to TIO within 26 weeks but then got inferior after 39 weeks. On one hand, the disproportionate number of poor performers who discontinued TIO during the final 3 months of treatment (Donohue et al., 2019b) could partially account for this phenomenon. On the other hand, the distinct mechanism of drug action should also be considered, as REV exhibits pharmacological effects through selective inhibition of M_3_ receptor at the smooth muscle leading to bronchodilation, while TIO blocks both M_3_ and M_1_ receptors to take more prolonged effects (Li and Yang, 2019). Given that REV with novel biphenyl carbamate tertiary amine structure is different from TIO with quaternary ammonium feature (Donohue et al., 2019d; Montuschi and Ciabattoni, 2015), REV was supposed to have higher metabolic lability and more rapid systemic clearance than TIO (Babu and Morjaria, 2017; GlaxoSmithKline. Incruse, 2021) in terms of minimizing systemically mediated AEs. Nonetheless, this systematic review did not show any significant advantage of REV in reducing the risk of AEs or SAEs compared to TIO or IPR, which might be ascribed to underpowered sample size. Although present evidence showed that REV was not preferable to TIO both in efficacy and safety, certain COPD patients with chronic muscle weakness, or cognitive or visual impairment or diminished manual dexterity may still particularly benefit from the use of this once-daily nebulized delivery LAMA (Bonini and Usmani, 2015; Tashkin D. P., 2016). As the evidence about the efficacy and safety of REV vs. TIO was mainly from two trials (NCT02518139 and NCT03095456) with high risk of attribution, reporting, and other bias, its confidence rating was graded as very low to low quality.

This systematic review also found the therapeutical course would influence the efficacy in improving trough FEV_1_, which could be explained by the progression of COPD with longer follow-up time. Considering the limited data from trials, we did not evaluate the association of reduced efficacy with treatment course. Furthermore, the proportion of ICS/LABA users varied a lot among all the included trials, which probably brought heterogeneity to the results of meta-analyses. Nevertheless, a subgroup analysis (Sethi et al., 2020) found that REV produced similar improvements from baseline in trough FEV_1_ in the non-LABA and LABA groups despite more AEs reported in the LABA.

There are several limitations in this study. As we only included RCTs, the results may not have good generalizability for strict inclusion criteria and small sample size. Particularly, the representativeness of participants was compromised because all the trials were conducted in the United States, the United Kingdom, Northern Ireland, New Zealand, and South Africa where most of the participants were white. In addition, these trials were not sensitive to assess treatment-related rare AEs (incidence ≤0.01%) due to relatively lower power of test and shorter follow-up term. Furthermore, the quality of evidence was subpar for the high risk of attribution and reporting bias in primary studies. Moreover, the language restriction for English and Chinese could also reduce the generalizability of our results. Therefore, prospective, multicenter, RCTs with larger samples, different populations, and better methodological design are urgently needed in this field. Although the course of treatment would influence the efficacy of REV, we performed the dose-response meta-analysis without adjusting this confounder due to limited data from the trials, suggesting that the non-linearity relationship between dosage and improvements in the through FEV_1_ of REV should be interpreted with caution. Finally, even though study design and concomitant medication such as formoterol in NCT03573817 would also be the possible source of heterogeneity, we did not assess the effect of these factors on the results due to small quantity of trials with the same outcomes.

To conclude, based on the findings of our systematic review and dose-response meta-analysis of RCTs, REV appears to be a promising option for the treatment of moderate to very severe COPD. Considering the low confidence rating of evidence, further studies are warranted to compare the efficacy, long-term safety and cost-effectiveness between REV and other LAMAs (TIO) in different populations. Although most studies used the FEV_1_ to evaluate the efficacy of REV in treatment of COPD, but FEV_1_ should just be set as a surrogate outcome. Therefore, the clinical benefit of REV in patients with COPD should be further evaluated. And researchers should increase focus on those important endpoints (e.g., death, exacerbations requiring antibiotics or oral steroids, hospitalizations due to exacerbation of COPD, exacerbations requiring a short course of an oral steroid or antibiotic, etc.) and patient-reported outcomes in the further research due to few trials reporting such related endpoints.

## Data Availability

The original contributions presented in the study are included in the article/Supplementary Material, further inquiries can be directed to the corresponding author.
