# Supplementary material for: The Efficacy and Safety of Revefenacin for the Treatment of Chronic Obstructive Pulmonary Disease: A Systematic Review
Source: Front Pharmacol. 2021 Oct 20;12:667027. doi: 10.3389/fphar.2021.667027 (PMC8564370; doi:10.3389/fphar.2021.667027)
Supplement: Supplementary file 1 [file DataSheet1.docx]

**Table S1 PRISMA 2009 Checklist**

| **Section/topic** | **#** | | **Checklist item** | | **Reported on page #** |
| --- | --- | --- | --- | --- | --- |
| **TITLE** | | | | |  |
| Title | 1 | | Identify the report as a systematic review, meta-analysis, or both. | | Page 1 |
| **ABSTRACT** | | | | |  |
| Structured summary | 2 | | Provide a structured summary including, as applicable: background; objectives; data sources; study eligibility criteria, participants, and interventions; study appraisal and synthesis methods; results; limitations; conclusions and implications of key findings; systematic review registration number. | | Page 3  (PROSPERO, CRD42020182793) |
| **INTRODUCTION** | | | | |  |
| Rationale | 3 | | Describe the rationale for the review in the context of what is already known. | | Page 4 |
| Objectives | 4 | | Provide an explicit statement of questions being addressed with reference to participants, interventions, comparisons, outcomes, and study design (PICOS). | | Page 4 to 5 |
| **METHODS** | | | | |  |
| Protocol and registration | 5 | | Indicate if a review protocol exists, if and where it can be accessed (e.g., Web address), and, if available, provide registration information including registration number. | | (PROSPERO, CRD42020182793) |
| Eligibility criteria | 6 | | Specify study characteristics (e.g., PICOS, length of follow-up) and report characteristics (e.g., years considered, language, publication status) used as criteria for eligibility, giving rationale. | | Page 5 to 6 |
| Information sources | 7 | | Describe all information sources (e.g., databases with dates of coverage, contact with study authors to identify additional studies) in the search and date last searched. | | Page 5 |
| Search | 8 | | Present full electronic search strategy for at least one database, including any limits used, such that it could be repeated. | | Table S2 |
| Study selection | 9 | | State the process for selecting studies (i.e., screening, eligibility, included in systematic review, and, if applicable, included in the meta-analysis). | | Page 6 |
| Data collection process | 10 | | Describe method of data extraction from reports (e.g., piloted forms, independently, in duplicate) and any processes for obtaining and confirming data from investigators. | | Page 6 |
| Data items | 11 | | List and define all variables for which data were sought (e.g., PICOS, funding sources) and any assumptions and simplifications made. | | Page 6 |
| Risk of bias in individual studies | 12 | | Describe methods used for assessing risk of bias of individual studies (including specification of whether this was done at the study or outcome level), and how this information is to be used in any data synthesis. | | Page 6 |
| Summary measures | 13 | | State the principal summary measures (e.g., risk ratio, difference in means). | | Page 7 |
| Synthesis of results | 14 | | Describe the methods of handling data and combining results of studies, if done, including measures of consistency (e.g., I^2^) for each meta-analysis. | | Page 7 |
| Risk of bias across studies | | 15 | | Specify any assessment of risk of bias that may affect the cumulative evidence (e.g., publication bias, selective reporting within studies). | Page 7 |
| Additional analyses | | 16 | | Describe methods of additional analyses (e.g., sensitivity or subgroup analyses, meta-regression), if done, indicating which were pre-specified. | Page 7 |
| **RESULTS** | | | | |  |
| Study selection | | 17 | | Give numbers of studies screened, assessed for eligibility, and included in the review, with reasons for exclusions at each stage, ideally with a flow diagram. | Page 8.  Figure 1 |
| Study characteristics | | 18 | | For each study, present characteristics for which data were extracted (e.g., study size, PICOS, follow-up period) and provide the citations. | Page 8  Table 1 |
| Risk of bias within studies | | 19 | | Present data on risk of bias of each study and, if available, any outcome level assessment (see item 12). | Page 8 to 9  Figure 2 |
| Results of individual studies | | 20 | | For all outcomes considered (benefits or harms), present, for each study: (a) simple summary data for each intervention group (b) effect estimates and confidence intervals, ideally with a forest plot. | Page 9 to 11  Figure 4 and 5  Table 2  Figure S3 |
| Synthesis of results | | 21 | | Present results of each meta-analysis done, including confidence intervals and measures of consistency. | Page 9 to 11  Figure 4 to 5  Table 2 to Table 5  Figure S3 |
| Risk of bias across studies | | 22 | | Present results of any assessment of risk of bias across studies (see Item 15). | Page 9 to 11  Table 3 |
| Additional analysis | | 23 | | Give results of additional analyses, if done (e.g., sensitivity or subgroup analyses, meta-regression [see Item 16]). | Page 9 to 11  Figure 3  Table 2  Figure S1, S2, S4, S5, and S6 |
| **DISCUSSION** | | | | |  |
| Summary of evidence | | 24 | | Summarize the main findings including the strength of evidence for each main outcome; consider their relevance to key groups (e.g., healthcare providers, users, and policy makers). | Page 11 to 13 |
| Limitations | | 25 | | Discuss limitations at study and outcome level (e.g., risk of bias), and at review-level (e.g., incomplete retrieval of identified research, reporting bias). | Page 13 |
| Conclusions | | 26 | | Provide a general interpretation of the results in the context of other evidence, and implications for future research. | Page 13 |
| **FUNDING** | | | | |  |
| Funding | | 27 | | Describe sources of funding for the systematic review and other support (e.g., supply of data); role of funders for the systematic review. | National Natural Science Foundation of China (No. 72064004) and Doctoral Foundation of Guizhou Provincial People’s Hospital (GZSYBS [2019] No.09). The funder of the study did not participate in study design, data collection, data analysis, data interpretation, or writing of the report. |

**Table S2. Searching Strategy**

| **Database** | **Search Strategy** | **Results** |
| --- | --- | --- |
| PubMed | #1 Revefenacin [Mesh]  #2 Revefenacin [Title/Abstract]  #3 Revefenacin  #4 TD-4208 [Mesh]  #5 TD-4208 [Title/Abstract]  #6 TD-4208  #7 Yupelri [Mesh]  #8 Yupelri [Title/Abstract]  #9 Yupelri  #10 #1~#9 OR | 33 |
| EMbase | #1 ‘Revefenacin’ /exp  #2 Revefenacin: ab, ti  #3 Revefenacin  #4 ‘TD 4208’ /exp  #5 ‘TD 4208’: ab, ti  #6 ‘TD 4208’  #7 ‘Yupelri’ /exp  #8 Yupelri: ab, ti  #9 Yupelri  #10 #1~#9 OR | 79 |
| Cochrane | #1 Revefenacin: ti, ab, kw  #2 TD-4208 : ti, ab, kw  #3 Yupelri: ti, ab, kw  #4 #1~#3 OR | 51 |

**Table S3. The results of the pairwise meta-analysis of change from baseline in trough FEV_1_ (omitting crossover studies)**

| **Group** | **Follow-up time** | ***N*** | ***n*** | **Heterogeneity** | **Model** | ***MDs(ml)*** | **95%*CIs*** | ***P*** |
| --- | --- | --- | --- | --- | --- | --- | --- | --- |
| REV 44 vs PLA | 4 weeks | 1 | 60 vs. 55 | *NA* | *NA* | 51.80 | [42.59, 61.01] | <0.00001 |
| REV 88 vs PLA  REV 175 vs PLA  REV 350 vs PLA | 4 weeks | 1 | 63 vs. 55 | *NA* | *NA* | 187.40 | [178.35, 196.45] | <0.00001 |
|  | 12 weeks | 1 | 161 vs. 146 | *NA* | *NA* | 79.22 | [75.72, 82.72] | <0.00001 |
|  | 12 weeks | 1 | 152 vs. 150 | *NA* | *NA* | 160.50 | [156.27, 164.73] | <0.00001 |
|  | 4 weeks | 1 | 59 vs. 55 | *NA* | *NA* | 166.60 | [157.33, 175.87] | <0.00001 |
|  | 12 weeks | 2 | 310 vs. 296 | *I^2^*=0%, *P*=0.58 | Fixed | 146.91 | [144.20, 149.63] | <0.00001 |
|  | 4 weeks | 1 | 63 vs. 55 | *NA* | *NA* | 170.60 | [161.59, 179.61] | <0.00001 |
| REV 88 vs TIO | 4 weeks | 1 | 317 vs. 330 | *NA* | *NA* | -29.00 | [-30.82, -27.18] | <0.00001 |
|  | 13 weeks | 1 | 287 vs. 307 | *NA* | *NA* | -16.00 | [-17.96, -14.04] | <0.00001 |
|  | 26 weeks | 1 | 239 vs. 283 | *NA* | *NA* | -14.80 | [-16.99, -12.61] | <0.00001 |
|  | 39 weeks | 1 | 223 vs. 265 | *NA* | *NA* | -10.20 | [-12.51, -7.89] | <0.00001 |
|  | 52 weeks | 1 | 212 vs. 248 | *NA* | *NA* | -42.70 | [-45.12, -40.28] | <0.00001 |
| REV 175 vs TIO | 4 weeks | 2 | 371 vs. 420 | *I^2^*=66%, *P*=0.08 | Random | 13.51 | [8.32, 18.69] | <0.00001 |
|  | 13 weeks | 1 | 243 vs. 307 | *NA* | *NA* | 2.70 | [0.55, 4.85] | <0.00001 |
|  | 26 weeks | 1 | 210 vs. 283 | *NA* | *NA* | 15.40 | [13.03, 17.77] | <0.00001 |
|  | 39 weeks | 1 | 189 vs. 265 | *NA* | *NA* | -8.30 | [-10.85, -5.75] | <0.00001 |
|  | 52 weeks | 1 | 185 vs. 248 | *NA* | *NA* | -39.20 | [-41.82, -36.58] | <0.00001 |


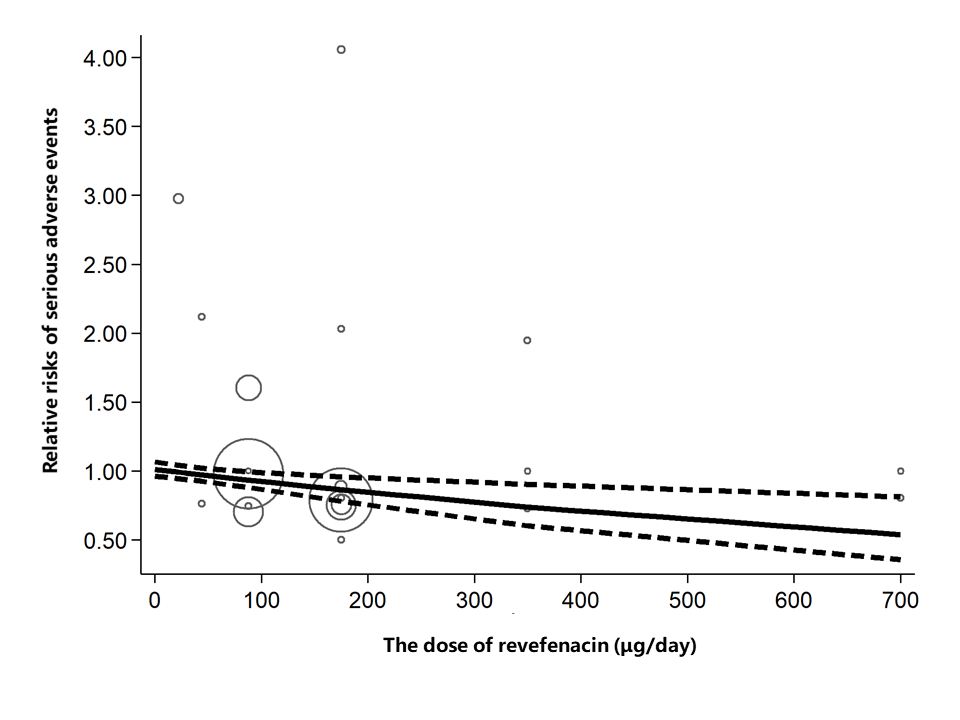


**Figure S1. Increase in dose (μg/day) of revefenacin and risk of total adverse events.** The solid line is the linear prediction of the risk and the dotted lines indicate the 95% confidence interval.


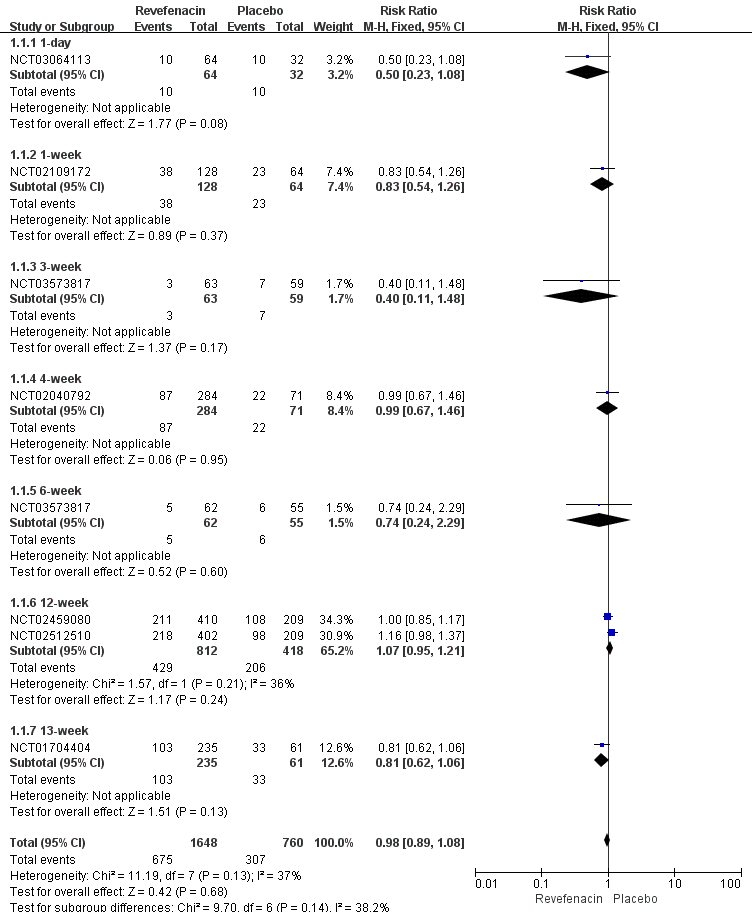


**Figure S2. Different therapeutic course subgroup analyses of total adverse events for revefenacin vs. placebo.**


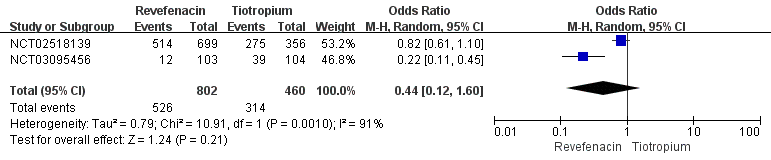


**Figure S3. Total adverse events for revefenacin vs. tiotropium in patients with chronic obstructive pulmonary disease.**


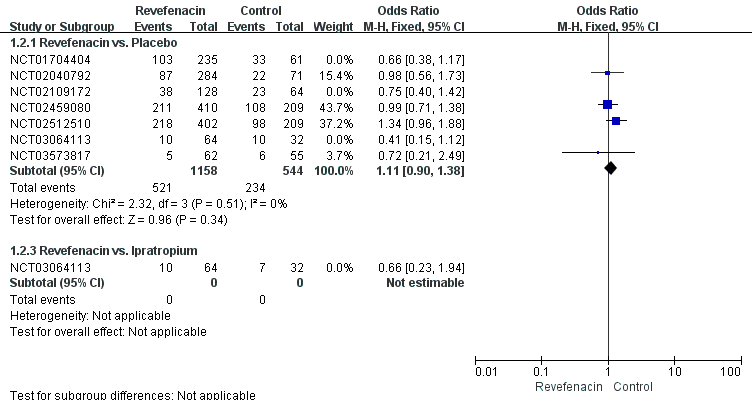


**Figure S4. The sensitivity analyses of any adverse events by omitting crossover studies.**


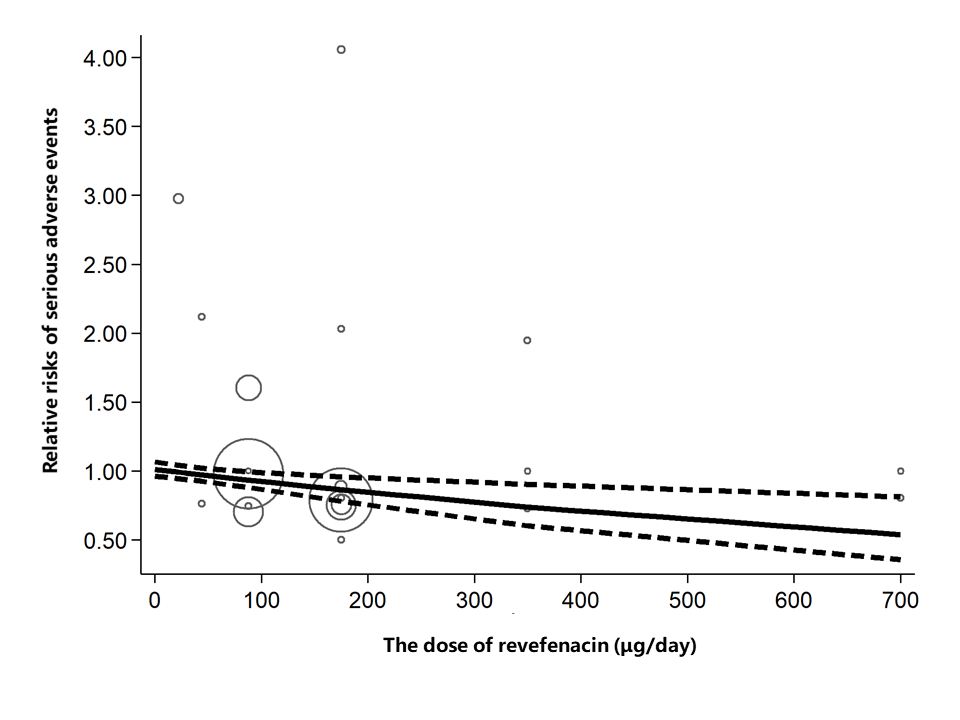


**Figure S5. Increase in dose (μg/day) of revefenacin and risk of serious adverse events.** The solid line is the linear prediction of the risk and the dotted lines indicate the 95% confidence interval.


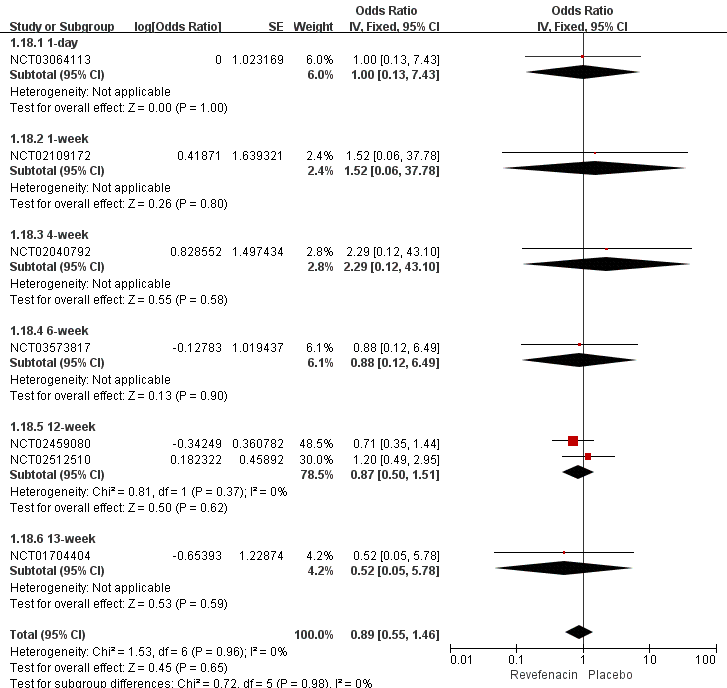


**Figure S6. Different therapeutic course subgroup analyses of serious adverse events for revefenacin vs. placebo.**


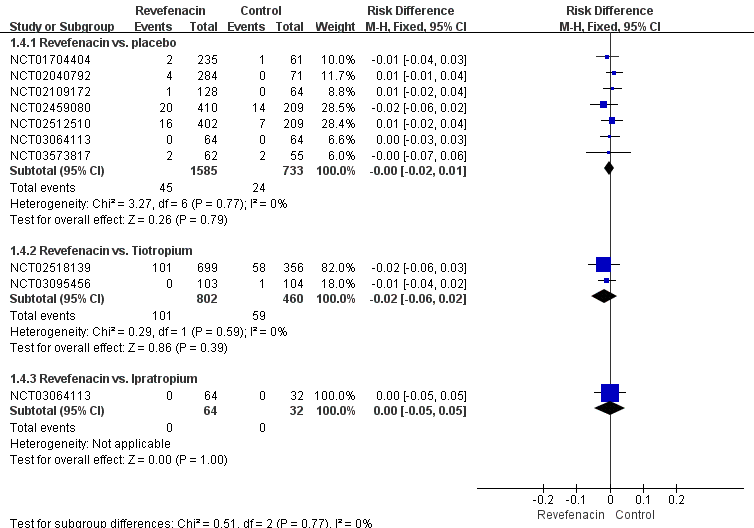


**Figure S7. The sensitivity analyses of serious adverse events by using Mantel-Haenszel risk difference.**


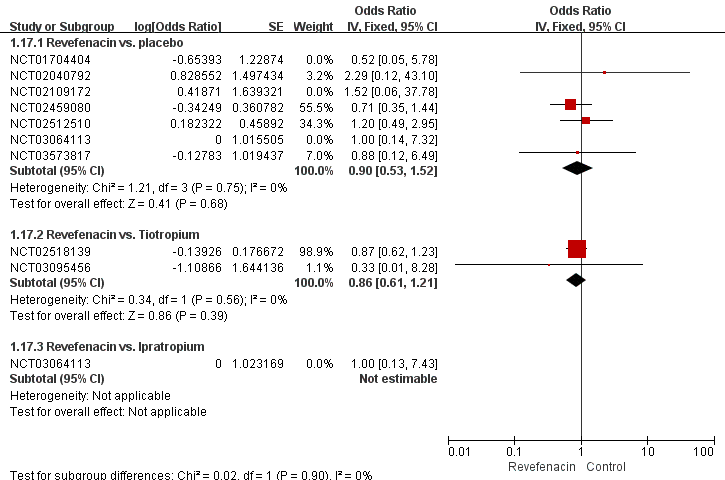


**Figure S8. The sensitivity analyses of serious adverse events by omitting crossover studies.**
